# Construction and validation of a predictive model for lower extremity deep vein thrombosis after total knee arthroplasty

**DOI:** 10.1097/MD.0000000000038517

**Published:** 2024-06-14

**Authors:** Qiang Peng

**Affiliations:** a Ya’an Hospital of Traditional Chinese Medicine.

**Keywords:** deep vein thrombosis, knee, nomogram, replacement

## Abstract

The aim was to investigate the independent risk factors for lower extremity deep vein thrombosis (DVT) after total knee arthroplasty, and to establish a nomogram prediction model accordingly. Data were collected from total knee replacement patients from January 2022 to December 2023 in our hospital. Unifactorial and multifactorial logistic regression analyses were used to determine the independent risk factors for lower extremity DVT after total knee arthroplasty and to establish the corresponding nomogram. The receiver operating characteristic curves were plotted and the area under the curve was calculated, and the calibration curves and decision curves were plotted to evaluate the model performance. A total of 652 patients with total knee arthroplasty were included in the study, and 142 patients after total knee arthroplasty developed deep veins in the lower extremities, with an incidence rate of 21.78%. After univariate and multivariate logistic regression analyses, a total of 5 variables were identified as independent risk factors for lower extremity DVT after total knee arthroplasty: age > 60 years (OR: 1.70; 95% CI: 1.23–3.91), obesity (OR: 1.51; 95% CI: 1.10–1.96), diabetes mellitus (OR: 1.80; 95% CI: 1.23–2.46), D-dimer > 0.5 mg/L (OR: 1.47; 95% CI: 1.07–1.78), and prolonged postoperative bed rest (OR: 1.64; 95% CI: 1.15–3.44). the nomogram constructed in this study for lower extremity DVT after total knee arthroplasty has good predictive accuracy, which helps physicians to intervene in advance in patients at high risk of lower extremity DVT after total knee arthroplasty.

## 1. Introduction

Total knee arthroplasty has now become one of the main treatments for end-stage osteoarthritis,^[1]^ showing good results in relieving joint pain and promoting functional recovery of the knee.^[2–4]^ Deep vein thrombosis (DVT) is a common perioperative complication of total knee arthroplasty,^[5]^ especially in the lower limbs. If it is not controlled in time, it can lead to deep vein insufficiency or even disability; and once pulmonary embolism occurs, the morbidity and mortality rate is more than 30%.^[6,7]^ Therefore, it is particularly important to explore the factors affecting lower extremity DVT after total knee arthroplasty, to identify the risk group of lower extremity DVT after surgery, and to actively correct the reversible triggers. The lack of specific symptoms in the early stage of DVT after total knee arthroplasty^[8]^ makes it difficult to accurately predict the risk of DVT by traditional laboratory indicators,^[9]^ and there is less evidence of relevant predictive models. Nomogram have been used in many fields, not only for the prediction of post-fracture lower extremity DVT,^[10]^ but also for the prediction of post-fracture pneumonia^[11]^ and post-fracture delirium,^[12]^ etc, and all of them have achieved good prediction results. The aim of the present study was to investigate the independent risk factors for lower extremity DVT after total knee arthroplasty and to establish a nomogram prediction model accordingly.

## 2. Information and methods

### 2.1. Data sources and data collection

This study retrospectively analyzed the data of patients hospitalized for total knee replacement from January 2022 to December 2023 in our hospital. The study was retrospective and complied with the relevant standards of the Hospital Ethics Committee.

### 2.2. Inclusion and exclusion criteria

Inclusion criteria: diagnosis of osteoarthritis of the knee in all cases; treatment with total knee arthroplasty; completion of venous ultrasound screening for venous thrombosis of the lower extremities during hospitalization; complete clinical data. Exclusion criteria: history of previous knee surgery; combination of serious cardiovascular and cerebrovascular diseases; combination of malignant tumors; previous history of lower extremity DVT.

### 2.3. Collection of relevant variables

Relevant information of patients with knee osteoarthritis was collected, including patients’ age, gender, body mass index (BMI), smoking, alcohol consumption, hypertension, diabetes mellitus, hyperlipidemia, coronary heart disease, varicose veins of the lower limbs, tourniquet use time, intraoperative blood loss, postoperative prothrombin time, postoperative thrombin time, postoperative fibrinogen, postoperative D-dimer, and postoperative bed rest time.

### 2.4. Statistical analysis

The collected data were randomly divided into modeling group (70%) and validation group (30%) in the ratio of 7:3 in R (4.2.1) software. In the modeling group, SPSS26.0 software was used for one-way analysis of the differences between the 2 groups, and the chi-square test was used to statistically analyze the count data. Variables with *P* < .05 screened by unifactorial analysis were included in multifactorial logistic regression analysis, and variables with *P* < .05 in multifactorial logistic regression analysis were determined to be independent risk factors for lower extremity DVT after total knee arthroplasty. The independent risk factors selected in the multifactorial logistic regression analysis were plotted as nomograms in R software, and the receiver operating characteristic curves were plotted and the area under the curve was calculated in the modeling and validation groups, and the calibration curves and decision curves were plotted to evaluate the model performance.

## 3. Results

### 3.1. General situation

A total of 652 patients who underwent total knee arthroplasty were included in this study, and a total of 142 patients after total knee arthroplasty developed deep veins in the lower extremities, with an incidence rate of 21.78%. According to the ratio of 7:3, 456 and 196 patients were randomly divided into modeling group and validation group.

### 3.2. Independent risk factors for lower extremity DVT after total knee arthroplasty

In the modeling group, 17 variables were analyzed by univariate logistic regression analysis, and the results showed that 11 variables were potential risk factors for lower extremity DVT after total knee arthroplasty, including age, BMI, smoking, diabetes mellitus, hyperlipidemia, varicose veins of the lower extremity, time of tourniquet use, postoperative prothrombin time, postoperative thrombin time, postoperative D-dimer, and postoperative bedtime (Table 1). Age > 60 years (OR: 1.70; 95% CI: 1.23–3.91), obesity (OR: 1.51; 95% CI: 1.10–1.96), diabetes mellitus (OR: 1.80; 95% CI: 1.23–2.46), and D-dimer > 0.5 mg/L (OR: 1.47; 95% CI: 1.07–1.78), and prolonged postoperative bed rest (OR: 1.64; 95% CI: 1.15–3.44) were independent risk factors for lower extremity DVT after total knee arthroplasty (Table 2).

**Table 1:** Univariate analysis of lower limb deep vein thrombosis after total knee arthroplasty.

Risk factor	Thrombosis group(n = 100)	Nonthrombotic group (n = 356)	*P*
Age			.000
≤60 yr	22	170	
>60 yr	78	186	
Sex			.501
Male	42	163	
Female	58	193	
BMI			.005
≤24 kg/m^2^	34	178	
>24 kg/m^2^	66	178	
Smoking			.032
Yes	38	96	
No	62	270	
Drinking			.721
Yes	43	146	
No	57	210	
Hypertension			.219
Yes	72	233	
No	28	123	
Diabetes			.002
Yes	69	184	
No	31	171	
Hyperlipidemia			.043
Yes	47	128	
No	53	228	
Coronary heart disease			.557
Yes	36	117	
No	64	239	
Varicose veins in the lower limbs			.044
Yes	16	32	
No	84	324	
Tourniquet use time			.035
≤90 mine	44	199	
>90 mine	56	157	
Intraoperative blood loss			.294
≤50 mL	59	189	
>50 mL	41	167	
Postoperative prothrombin time			.041
≤14 s	39	180	
>14 s	61	176	
Postoperative thrombin time			.017
≤16 s	36	176	
>16 s	64	180	
Postoperative fibrinogen			833
≤4 g/L	46	168	
>4 g/L	54	188	
Postoperative D-dimer			.009
≤0.5 mg/L	32	166	
>0.5 mg/L	68	190	
Postoperative bedtime			.017
≤24 h	33	165	
>24 h	67	191	

BMI = body mass index.

**Table 2:** Multifactorial analysis of lower extremity deep vein thrombosis after total knee arthroplasty.

Factors	β	SE	Wald χ^2^	*P*	OR	95%CI
Age > 60 yr	0.34	0.20	6.95	.041	1.70	1.23~3.91
BMI > 24 kg/m^2^	3.69	1.33	9.10	.024	1.51	1.10~1.96
Diabetes	0.14	0.09	10.95	.016	1.80	1.23~2.46
D-dimer > 0.5 mg/L	1.37	0.66	14.57	.000	1.47	1.07~1.78
Bedtime > 24 h	0.21	0.18	21.54	.000	1.64	1.15~3.44

BMI = body mass index.

### 3.3. Nomogram development and validation

**Figure 1.**
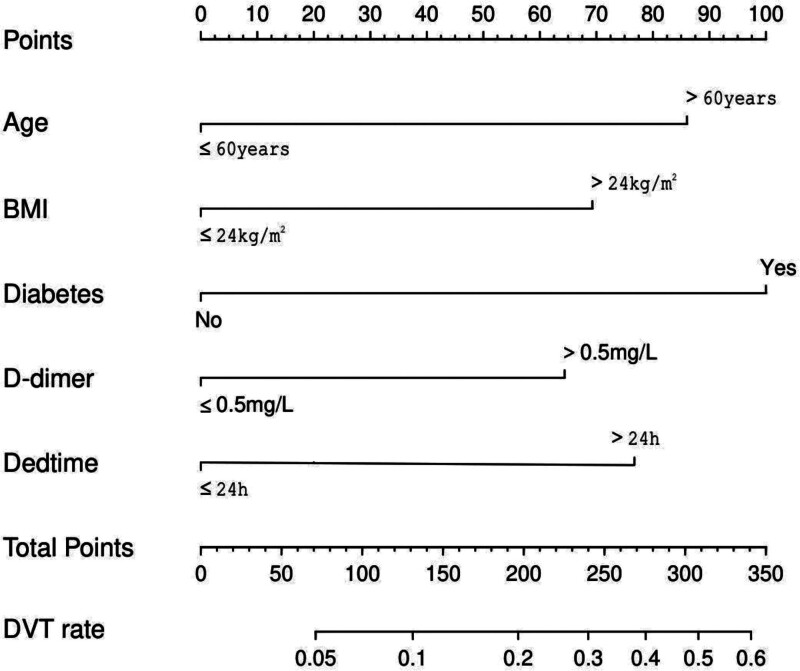
Nomogram prediction model for lower extremity deep vein thrombosis after total knee arthroplasty.

**Figure 2.**
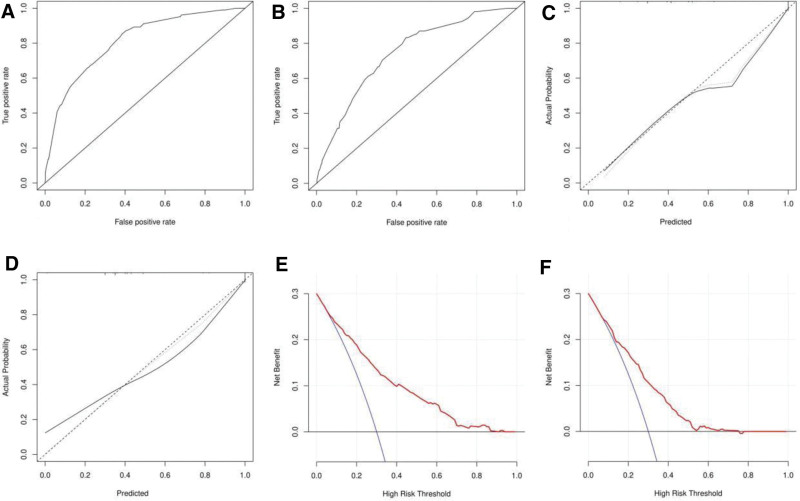
ROC curves of the nomograms in the training set (A) and validation set (B) for predicting lower extremity DVT after total knee arthroplasty. Calibration curves of nomograms for predicting lower extremity DVT after total knee replacement in training set (C) and validation set (D). Decision curves for the nomograms used to predict lower extremity deep vein thrombosis after total knee arthroplasty in training set (E) and validation set (F). DVT = deep vein thrombosis.

A nomogram was drawn using the screened independent risk factors and used to predict the risk of lower extremity DVT after total knee arthroplasty (Fig. 1). ROC curves were then plotted for the modeling and validation groups, and the corresponding areas under the curve were calculated to be 0.811 and 0.743 (Fig. 2A and B). In addition, calibration curves were plotted, indicating that the nomogram-predicted risk was in good agreement with the actual occurrence risk and had good predictive ability (Fig. 2C and D). Also, the decision curve showed that nomogram had good predictive ability (Fig. 2E and F).

## 4. Discussion

In recent years, total knee arthroplasty has been widely used in clinical practice, and the factors associated with postoperative lower extremity DVT and its impact on clinical prognosis have received increasing attention.^[13,14]^ Several previous studies have explored the risk factors for postoperative DVT after total knee arthroplasty, but the conclusions varied between studies, and data on relevant predictive models are still lacking.^[15–17]^ In the present study, we used predictors that are common and easily recognized in clinical practice, and developed this nomogram prediction model based on the 5 independent risk factors identified, and after model validation, it was determined that the model established in this study had good predictive ability.

In this study, advanced age was found to be a risk factor for lower extremity DVT after total knee arthroplasty. It has been shown^[18,19]^ that elderly patients have degenerative changes in vascular elasticity due to the decline in blood circulation function, leading to a subsequent decrease in the body ability to dissolve fibrin, slowing down or even stagnation of blood flow, which is thought to be closely related to the occurrence of DVT. Therefore, increased attention should be paid to elderly patients.

In this study, obesity was found to be a risk factor for lower extremity DVT after total knee arthroplasty. It has been reported^[20]^ that the blood flow rate after total knee arthroplasty is slower in obese patients than in people with normal weight, and the risk of postoperative DVT is higher in the population with a BMI of more than 28 kg/m^2^. One study^[21]^ noted that blood viscosity is usually higher in obese patients, which increases the likelihood of thrombosis. Secondly, obesity may lead to a chronic inflammatory state that affects the balance of blood coagulation and fibrinolytic systems. In addition, obese patients have limited mobility and slow recovery after surgery, which leads to slower venous blood flow to the lower extremities, increasing the risk of lower extremity DVT.^[22]^ The results of study^[23]^ also showed that preoperative weight loss significantly reduced the risk of postoperative lower extremity DVT, so it is recommended that obese patients lose as much weight as possible before surgery.

In this study, having diabetes mellitus was found to be a risk factor for lower extremity DVT after total knee arthroplasty. One study^[24]^ found that diabetic patients are often associated with a hyperglycemic state, which can lead to vascular endothelial cell damage and dysfunction, thereby promoting thrombosis. In addition, diabetic patients have increased platelet aggregation and blood viscosity, further increasing the risk of thrombosis.^[25]^ Diabetes mellitus can also cause microangiopathy and vascular inflammation, and these pathological changes may lead to abnormal blood rheology and affect normal circulation.^[26]^ Therefore, blood glucose levels are strictly controlled preoperatively and postoperatively to reduce vascular damage and blood coagulation tendency due to hyperglycemia.

In this study, elevated postoperative D-dimer was found to be a risk factor for lower extremity DVT after total knee arthroplasty. D-dimer is a specific small protein fragment produced by fibrin degradation and is often used as a biochemical marker of thrombosis.^[27]^ Studies have suggested that elevated D-dimer levels after total knee arthroplasty usually reflect the presence of active thrombosis and subsequent fibrinolytic activity in the body.^[28]^ Elevated postoperative D-dimer levels are closely associated with the formation of DVT in the lower extremities, which may be due to vascular injury during surgery, hemodynamic changes, and postoperative inflammatory response.^[29]^ For patients with significantly elevated D-dimer levels, signs of thrombosis should be closely monitored, and the need for further imaging to confirm the diagnosis of lower extremity DVT should be considered on a case-by-case basis. Secondly, pharmacologic prophylaxis, such as low molecular weight heparin or new oral anticoagulants, can be used to inhibit thrombosis based on patient-specific conditions and risk assessment.

In this study, prolonged postoperative bed rest was found to be a risk factor for lower extremity DVT after total knee arthroplasty. It was found that prolonged bed rest reduces the activity of leg muscles, which in turn reduces the venous return rate and increases the time blood remains in the veins, thus promoting thrombus formation.^[30]^ In addition, surgery itself causes damage to the vascular endothelium and an inflammatory response, factors that likewise contribute to thrombosis.^[31]^ Therefore, rehabilitation training should be started as early as possible after surgery, and patients should be encouraged to perform moderate lower limb activities under the guidance of healthcare professionals to promote blood circulation. Secondly, physical therapy such as intermittent air pressure pump or compression stockings can be considered to assist the return of blood to the legs and reduce the possibility of thrombosis.

However, there are also shortcomings in this study. Firstly, as this is a retrospective study, there will be some unavoidable errors arising. Second, this was a single-center study conducted in a tertiary care hospital, and there was a bias in the selection of patients due to the admission of patients with more severe or complex conditions. Thirdly, this is a risk prediction model developed in a single center and therefore its validity needs to be validated in further multicenter studies.

## 5. Conclusion

The results of this study suggest that advanced age, obesity, diabetes mellitus, elevated postoperative D-dimer and prolonged postoperative bed rest are risk factors for lower extremity DVT after total knee arthroplasty. The nomogram constructed in this study for lower extremity DVT after total knee arthroplasty has good predictive accuracy, which helps physicians to intervene in advance in patients at high risk of lower extremity DVT after total knee arthroplasty.

## Acknowledgments

I would like to thank Deng Guanghua for his guidance in analyzing the data and submitting my thesis.

## Author contributions

**Conceptualization:** Qiang Peng.

**Data curation:** Qiang Peng.

**Formal analysis:** Qiang Peng.

**Funding acquisition:** Qiang Peng.

**Investigation:** Qiang Peng.

**Methodology:** Qiang Peng.

**Project administration:** Qiang Peng.

**Resources:** Qiang Peng.

**Software:** Qiang Peng.

**Supervision:** Qiang Peng.

**Validation:** Qiang Peng.

**Visualization:** Qiang Peng.

**Writing – original draft:** Qiang Peng.

**Writing – review & editing:** Qiang Peng.
